# Proton therapy in the treatment of hepatocellular carcinoma

**DOI:** 10.3389/fonc.2022.959552

**Published:** 2022-08-08

**Authors:** Francesco Dionisi, Daniele Scartoni, Francesco Fracchiolla, Irene Giacomelli, Benedetta Siniscalchi, Lucia Goanta, Marco Cianchetti, Giuseppe Sanguineti, Alberto Brolese

**Affiliations:** ^1^ Department of Radiation Oncology, IRCCS Regina Elena National Cancer Institute, Rome, Italy; ^2^ Proton Therapy Unit, Azienda Provinciale per i Servizi Sanitari, Trento, Italy; ^3^ Department of Advanced Biomedical Sciences, University of Naples “Federico II”, Napoli, Italy; ^4^ General Surgery & Hepato-Pancreato-Biliary Unit, Azienda Provinciale per i Servizi Sanitari, Trento, Italy

**Keywords:** proton therapy, hepatocellular carcinoma, active scanning, photon therapy, review

## Abstract

Liver cancer represents one of the most common causes of death from cancer worldwide. Hepatocellular carcinoma (HCC) accounts for 90% of all primary liver cancers. Among local therapies, evidence regarding the use of radiation therapy is growing. Proton therapy currently represents the most advanced radiation therapy technique with unique physical properties which fit well with liver irradiation. Here, in this review, we aim to 1) illustrate the rationale for the use of proton therapy (PT) in the treatment of HCC, 2) discuss the technical challenges of advanced PT in this disease, 3) review the major clinical studies regarding the use of PT for HCC, and 4) analyze the potential developments and future directions of PT in this setting.

## Introduction

Liver cancer represents the sixth most commonly diagnosed cancer and the third leading cause of cancer death worldwide in 2020 (1). Hepatocellular carcinoma (HCC) accounts for 90% of all liver cancers (2). Survival is poor with an average 5-year overall survival (OS) of around 20% (2). A certain level of cirrhosis is associated with the majority of HCCs. Locoregional recurrent disease is the main cause of death in HCC (3), while metastatic spread is limited even in advanced stages (4). Potential treatment strategies are extensive, ranging from curative approaches such as surgery or liver transplantation, which only selected patients (less than 20%) are eligible due to patients’ or tumors’ condition and ablation, whose efficacy is limited by tumor size or location (5), to non-curative strategies with an impact on survival for intermediate stages such as chemoembolization (6) or radioembolization (7). Advanced stages with retained liver function could benefit from systemic therapy: the recent IMbrave150 phase III trial showed the superiority of the combination of atezolizumab and bevacizumab over the standard of care treatment with sorafenib (8, 9).

In the context of localized diseases, radiotherapy (RT) represents a valid, curative, and alternative approach to surgery in various cancers (10). The case of HCC is more complex, due to several factors that historically limited the safety and efficacy of RT, such as the low radiotolerance of the liver, the need for high doses and treatment volumes of radiation for tumor control, the often impaired liver function at baseline, the impact of previous oncological treatments on liver status, and the lack of standard indications regarding the proper integration of RT with the abovementioned therapies.

However, nowadays, modern RT technologies allow safe HCC treatments with positive results in terms of local control (LC) and survival (11–13); thus, the role of RT in the treatment of HCC is slowly but steadily emerging as an effective locoregional treatment option for this disease according to several (but not all) international guidelines (14).

Proton therapy (PT) represents the most advanced radiotherapy technique currently available. In the past decade, PT registered an exponential rise in both the number of patients treated and the centers currently utilizing it worldwide, which exceeded 100 as of April 2022 (15). The unique physical properties of protons of having a finite range in tissue and a zero dose beyond the end of their path allow a dose-distribution profile which results in better sparing of healthy tissues compared with X-ray therapy at medium–low doses. These dosimetric characteristics fit well with liver irradiation and could enlarge the therapeutic window of RT in HCC treatment.

In this paper, we aim to review the rationale, the major clinical studies, the technical and economic challenges, and the potential future directions regarding the use of PT for HCC.

## Protons vs. X-rays (from dosimetry to initial clinical data)

The pioneering work of the University of Michigan established the strong correlation between the mean liver dose and the risk of radiation liver toxicity (i.e., radiation-induced liver disease, RILD) (16). Baseline liver function is also a predictor of the risk of RILD (17). Several dosimetric studies established the superiority of PT dose distribution compared with photon RT in liver irradiation: in 2008, the work of Wang compared proton and photon plans for nine patients affected by primary liver tumors, showing a significant gain with PT plans in the majority of parameters with a 30% reduction in the mean liver dose resulting in a significant reduction in the risk of RILD (18). More recently, the University of Pennsylvania published a planning comparison analysis between proton and photon plans for different tumor sizes (ranging from 1 to 6 cm) and locations in the liver (four different locations: dome, caudal, left medial, and central liver), and a total of 48 plans were analyzed. Based on their analysis, the authors suggested the use of protons to preserve liver function for tumors larger than 3 cm in the dome and central locations; according to the results of their work, protons should also be considered for tumors larger than 5 cm in any location. Interestingly, 3 cm is usually the cutoff size used to consider a high risk of failure in case of ablation treatment.

Moving from *in-silico* data to initial clinical data, in recent years, an interesting research field focused on the analysis of the clinical outcomes of HCC patients treated with PT in comparison with conventional photon RT. In 2015, Qi et al. reported the results of a meta-analysis of 70 clinical studies using particle therapy (including protons and carbon ion therapy) or X-rays for HCC (stereotactic RT, SBRT, or conformal 3D RT): a comparable efficacy in terms of OS and local control (LC) was found between particle therapy and SBRT, with a significant reduction in toxicity in favor of charged particles (19). More recently, in 2019, Sanford et al. analyzed the clinical outcomes of HCC patients treated with ablative PT (*n* = 49) or RT (*n* = 84) treated at the Massachusetts General Hospital between 2008 and 2017 (20). Treatment with PT was associated with significantly improved OS in comparison with RT (median OS 31 vs. 14 months), although there was no difference in LC (93% vs. 90% at 2 years). The authors hypothesized that this survival advantage was due to a lower incidence of liver decompensation with the use of protons. However, given the retrospective nature of the study, the authors warned regarding the risk of selection bias and invited to interpret the findings only as hypothesis generating. Similarly, a very recent work by Cheng et al. analyzed the outcome of HCC patients treated at their institution with PT (*n* = 64) or photon (*n* = 349) between 2007 and 2018 (21). In order to deal with the issue of selection bias for retrospective studies, the authors used the propensity score matching (PSM) method applied to predefined patient- and tumor-related variables, thus producing more reliable and robust clinical data regarding treatment comparison. A significant advantage in OS was reported in patients treated with PT in comparison with X-rays. Moreover, although the biologically effective dose (BED) was significantly higher in the PT population, the risk of RILD was significantly lower using protons.

The evidence provided thus far confirmed that the dosimetric gain achievable with PT in comparison with X-rays translates into an effective clinical benefit for HCC patients. The next step would be to demonstrate these clinical advantages in a randomized, controlled trial, which, albeit suffering from intrinsic weaknesses such as generality, duration, and costs (22), still represents the gold standard methodology to establish evidence of new medical therapies. This is the goal of the trial NCT03186898, a phase III randomized trial currently recruiting patients affected by unresectable HCC to determine whether OS is different between HCC patients treated with PT or X-rays.

## Proton vs. other treatment options

Among the several treatment options currently available for HCC treatment, the touchstone strategies for different disease stages are surgery, radiofrequency ablation, and chemoembolization (23). In light of this, it is necessary to analyze the studies which compared PT with such strategies.

Bush et al. from the Loma Linda proton center conducted a randomized trial comparing transarterial chemoembolization (TACE) and PT for HCC patients; so far, an interim analysis has been published showing similar OS between the two treatments and a trend with better LC (88% vs. 45%, *p* = .06) and progression-free survival (48% vs. 31%, *p* = .06) favoring PT (24). Tamura et al. recently reported the results of a retrospective comparison between surgery and PT for single HCC ≤100 mm without vessel invasion (25). The authors found that the median survival time in the surgery group was significantly better than in the PT group. The performance status (PS) of the patients was confirmed to be an independent prognostic factor for survival; as a matter of fact, the difference in OS between surgery and PT disappeared after PSM. In the context of PT treatment in comparison with other key strategies in HCC, the most important data come from the very recent phase III study by Kim et al. (26). The authors from Korea conducted a single-center, non-inferiority, randomized trial to compare PT vs. standard of care radiofrequency ablation (RFA) for HCC lesions <3 cm. The primary endpoint was 2-year local progression-free survival (LPFS). To our knowledge, for the first time, PT demonstrated a similar outcome in terms of efficacy in comparison with the gold standard treatment in a phase III randomized clinical trial. As a matter of fact, the 2-year LPFS with PT vs. RFA was 92.8% vs. 83.2% in the intention-to-treat population. As expected, the tolerability profile of PT was excellent, with no change in Child–Pugh score ≥2 points after PT treatment.

## Clinical studies

**Table 1** illustrates the major clinical studies regarding PT for HCC patients. In general, it is important to underline that the quantity of clinical data is high: PT indeed has been used to treat HCC since the 1980s, the first experience being reported in 1983 by the University of Tsukuba, Japan. Since that time, the data from thousands of HCC patients treated with PT have been published. In terms of the quality of the studies’ methodology, the majority of the reports represent institutional retrospective case series with a few prospective trials: of note, two randomized trials were published. The geographical distribution of the studies is also of note: the majority of the studies come from Eastern countries, where the incidence of HCC is the highest and where there is a high concentration of proton centers (15). The rest of the studies come from the USA, with only one retrospective series coming from the European countries (27). It is interesting to highlight that, in contrast to the European guidelines, the HCC guidelines from the USA and Asia suggest the use of PT as an effective alternative in the treatment of unresectable HCC in light of the clinical results reported in **Table 1**. As a matter of fact, all the studies reported positive results in terms of efficacy and safety for PT in HCC treatment. Going into detail, the comprehensive clinical experience of the University of Tsukuba assessed the effectiveness of PT for various clinical conditions of the tumor and patient. Three different treatment schedules were developed according to tumor location: lesions located adjacent to the porta hepatis (PH) and gastrointestinal (GI) tract were treated with prolonged schedules (72–77 Gy in 22–35 fractions), while lesions located ≥2 cm away from the GI tract received a more hypofractionated regimen of 66 Gy in 10 fractions. The reported 3- and 5-year OS rates were 64.7% and 44.6%, with a 5-year LC rate of 83.3%, respectively, without relevant toxicity (28). The same institution published other reports retrospectively analyzing the clinical data of a specific HCC population of patients such as patients with tumors larger than 10 cm, patients with portal vein invasion, elderly patients, and patients with poor liver function (Child–Pugh C) (29–32). Albeit retrospective, these case series illustrated the feasibility of PT in these challenging settings.

**Table 1:** Major clinical studies of PT and HCC.

Author, date	Center	Observation Period	Type of Study	Patient/Study CharacteristiCs	N° Patients	Median age	Performance status	Liver function (Child-Pugh)	Proton technique	Treatment Regimen Range	Equivalent dose 2 Gy/fr a/b = 10	tumor size(range)	median FUP (range)	LC	os	Toxicity≥g3
**Su et al , 2022**	Chang Gung Memorial Hospital, Taiwan	2016-2019	retrospective	PT combined with anti-PD1/PDL1	29	60	PS 0 16 pts, PS 1 13 pts	CP A5 23 pts, CP A6 6 pts	PSC	66 Gy/10 fr 3 pts 72.6 Gy /22 fr 18 pts 60 Gy/ 10 fr 2 pts 50 Gy/10 fr 2 pts 45 Gy/10 fr 1 pt 33 Gy/5 fr 2 pts 33 Gy/10 fr 1 pt	109.6 Gy_10_ 96.6 Gy_10_ 96 Gy_10_ 75 Gy_10_ 65.3 Gy_10_ 54.8 Gy_10_ 43.9 Gy_10_	≥ 5cm 19 pts	13 mo (1/48.1)	80% at 1 yr	63% at 2 yr	1 G3 dermatitis 1 G3 thrombocytopenia 3 G3 liver enzyme increase 4 G3 GI bleeding 1 G4 bilirubine increase 1 G4 biliary stricture 2 G5 hepatic failure 1 G5 duodenal perforation
**Lee et al, 2022**	Chang Gung Memorial Hospital, Taiwan	11/2015-02/2021	retrospective	unresectable HCC with bile duct invasion	20	61.5	PS 0 7 pts, PS 1 23 pts	CP A5 9 pts, CP A6 7 pts, CP B7 2 pts, CP B8 1 pts, CP B9 1 pts	2015-2016 PSC;2017-2022 PBS	72.6 Gy/22 fr	96.6 Gy_10_	6.3 cm (1.0–18.5)	19.9 mo (3.1-64.9)	1yr 94.7% ( 1yr cumulative local recurrence 5.3%)	1yr 79.4% 2yr 53.3%	3 G3 GI gastroduodenal ulcer 4 RILD
**Lin et al, 2021**	Chang Gung Memorial Hospital, Taiwan	2014 - 2017	retrospective	HCC patients without regional lymph node involvement or distant metastasis	43	71	PS 0 22 pts, PS 1 19 pts, PS 2 2 pts	CP A 40, CP B 3	PSC	25 pts 72.6 Gy/22 fr; 28pts 66Gy/10fr	96.6 Gy10; 109.6 Gy10	3.1 cm (1.1–17.1)	40 mo (9–62)	5yr 93.0%.	1yr 88.4%; 5yr 63.4%	1g3 skin 7 Child-Pugh score deterioration of 1 point.
**Bhangoo et al, 2021**	Mayo Clinic, USA	06/2015-12/2018	retrospective	all patients who were treated with IMPT for HCC with curative intent	37	69	PS 0 14 pts , PS 1 17 pts, PS 2 4, PS 3 2	CP A5-6 26 pts, CP B7-9 11 pts	PBS	15 pts 67.5 Gy /15 fr 13 pts 58.5 Gy /15 fr 15 pts 52.5 Gy /15 fr 6 pts 50 Gy /5 fr 37.5 Gy in 5 fx 1 (3%)	97.9 Gy10, 81.3 Gy10, 70.9 Gy10, 100 Gy10, 65.6 Gy10	5cm (3-8)	21 mo (17-30)	1yr 94%	1yr 78%	G3 pain Late 6pts increase CP by 2points
**Iwata et al, 2021**	Nagoya Proton Therapy Center	06/2013-12/2019	retrospective	elderly (≥80 years old) patients.	71	82	PS 0 44 pts, PS 1 20 pts, PS 2 4 pts, PS 3 3 pts	CP A5 49 pts, CP A6 15 pts, CP B7-9 7 pts	PSC	47 pts 66 Gy/10 Fr; 24pts 72.6 Gy/22 Fr	109.6 Gy10; 96.6 Gy10	32 mm (8–111)	33 mo (9–68)	2yr 88% (80–97%)	2yr 76% (66–87%)	1G3 dermatitis
**Dionisi et al, 2020**	Proton Therapy Unit, Trento	01/2018-12/2019	retrospective	unresectable disease	14	67	PS 0 11 pts, PS 1 5 pts, PS 2 2 pts	CP A5 10 pts, CP A6 2 pts, CP B7 1 pts	PBS	60Gy (50.31–67.5)	84 Gy10	4.5cm (1.2–13)	10mo (1-19)	100% at one year	63% at 1 y	/
**Kim TH et al, 2020**	National Cancer Center, Goyang, South Korea	03/2015-09/2018	prospective phase II	hypofractionated PBT in HCC	45	63	PS 0 45 pts	CP A 45 pts	PSC	70Gy/10fr	119 Gy10	1.6 cm (1.0–6.8)	35.1 mo (11.2–56.3)	3yr LPFS 95.2%	3yr 86.4%	Child–Pugh score showed a 1-point decrease in two patients (4.4%) and no change in 43 patients (95.6%)
**Parzen et al, 2020**	9 institutions in the USA	2013-2019	prospective registry	comparative efficacy of protons versus photons in patients with HCC.	30 HCC (63 total)	70.5	PS 0 13 pts, PS 1 10 pts, PS 2 5 pts, PS 3 1 pts	\	PSC or PBS	total population: 13pts 40 GyE (32.5–50)/5fr, 46pts 58.05 GyE (45–67.5)/15fr, 4pts 71.1 GyE (60.1–75)/25fr.	72 Gy10, 80.5 Gy10, 91.3 Gy10	4.3 cm (1.2–9.4)	8.2 mo	1yr 91.2%	1yr 71.5%	1G4 hyperbilirubinemia, 1G3 back pain.
**Yoo et al, 2020**	Samsung Medical Center, Seoul, Korea	01/2016-12/2017	retrospective	Evaluation of the risk of biliary complications after high-dose PBT for primary HCC	167	62	PS 0 92 pts, PS 1-2 75 pts	CP A 149 pts, CP B 15 pts, CP C 3 pts	PSC 130pts, PBS 37pts;	peripheral HCCs 66 Gy in 10 fractions hcc adjacent to the porta hepatis less than 1 cm, 72.6 Gy in 22 fractions	109.6 Gy10, 96.6 Gy10	peripheral 48pts <=2cm; 29pts >2cm; adjacent to the porta hepatis 26pts <=2cm, 54pts >2	14 months (range, 1–29 months	2ys infieldLC 86.5%	2yr OS 86.6%	2G 3 gastroduodenal ulcer 10 RILD 2 Non classic RILD
**Kim TH et al, 2019**	Center for Proton Therapy, Goyang, Korea	2012-2017	retrospective	PVTT	243	61	PS 0 237 pts, PS 1 6 pts	CP A 228 pts, CP B7 15 pts	NA	A=40pts PTV1 50 GyE/10 fr PTV2 30 GyE/10 fr; B=60pts PTV1 60 GyE /10 fr PTV2 30 GyE/10 fr; C=143pts PTV1=PTV2 66 GyE/10 fr	32.5 Gy10 /109.6 Gy10	all 2.2cm (1.0–17); A 6.0 cm (1.3–17), B 3.6 cm (1.0–12), C 1.5 (1.0–12.7cm )	31.5 mo (2.1–68.2)	3yr LRFS 88.6%; 5yr LRFS 87.5%; 5yr LFRS a 54.60% b 94.70% c 92.40%	3yr 61.8%, 5yr 48.1%. 5yr: a16.70%, b39.20%, c67.90%	Child–Pugh score 19 1-point decrease, 10 1-point increase, gastric or duodenal ulcers within the PBT field 1G1 3G2 1G3 GI in regimen A.
**Chadha et al, 2019**	MD Anderson Cancer Center	2007-2016	retrospective	HCC pts treated with PT	46	72	PS 0 (20%), PS 1 (25%), PS 2 (1%)	A 5 (26%) A6 (12%) B7 (6%) B8 (2%)	PSC	Median 67.5 GyE (24.0-91.0)/15 fr	81.6 Gy10, 67 Gy10, 73,2 Gy10	6 cm (1.5-21.0)	14.5 mo (0.4-59.8)	1yr 95%, 2yr 81%	1yr 73%, 2yr 62%	1 G3 diarrhea; 1 G3 erithema;, 4 G3 ascites 2G3 hipeblirubinema; 1G3 Upper gastrointestinal bleeding
**Shibata et al, 2018**	Proton Therapy Center, Fukui Prefectural Hospital, Japan	2011-2015	retrospective	effectiveness and toxicity of PT for hepatocellular carcinomas (HCC) >5 cm	29	71	PS 0: 21, PS 1: 7, PS 2: 1	A: 24; B:5	PBS	4 pts 66Gy/10fr; 13 pts 76Gy/20fr; 1 pts 80.5Gy/23fr; 1 pts 80Gy/25fr; 1 pts 67.5Gy/25fr; 5 pts 70.4Gy/32fr; 4 pts 76 Gy/38fr	91,3 Gy 87,4 Gy 90,6 Gy 88,5 Gy 71,4 Gy 71,6 Gy 76 Gy	6.9 Cm (50–139)	27 mo ( 2–72)	2yr & 4yr 95%	2yr 61%; 4yr 39%	ctcae 4.03 .. acute hyperbilirubinemia 1G3; Other patients had skin reactions Grade 2… late 6: pleural effusion 2G3, ascites 1G3, rib fracture 1G2, radiaton pneumonitis 1G2, erosions of ascending colon 1G2.
**Mizuhata et al, 2018**	Proton Therapy Center, Fukui Prefectural Hospital, Japan	03/2011-12/2015	retrospective	efficacy and toxicity of respiratory-gated PBT without fiducial markers for HCC located within 2 cm of the gastrointestinal tract.	40	72	PS 0,1: 38 \ PS 2: 2	A: 28; B: 12	PBS	1 pts 80.0 CGE/25fr 5 pts 76.0 CGE/38fr 17 pts 76.0 CGE/20fr 3 pts 74.8 CGE/34fr 8 pts 70.4 CGE/32fr 3 pts 70.0 CGE/35fr 1 pts 67.5 CGE/25fr 1 pts 66.0 CGE/30fr 1 pts 52.8 CGE/24fr	88 Gy 76 Gy 87,4 Gy 76,1 Gy 71,6 Gy 76 Gy 71,4 Gy 67,1 Gy 53,7 Gy	3.6 cm (1.1–12.4)	19.9 mo (1.2–72.3)	2yr 94%	1yr 86%; 2yr 76%	1 G3 gastric ulcer, 1 G3 ascites retention
**Lee et al, 2018**	Chang Gung Memorial Hospital, Taiwan	2015-2016	retrospective	HCC patients with small normal liver volume (NLV)	22	61,5	PS 0:7; PS 1: 13		PBS	median 72.6 GyE/22 fr	median 80,7 Gy_10_	5.3cm (1.2-15.0)	15.7 mo (4.0±24.9)	1yr 95.5%	1yr 91.8%	1G3 esophagitis 1G3 colitis; Child±Pugh score deterioration of one point 3 pts; 1G3 liver enzyme elevations
**Kim et al, 2018**	National Cancer Center, Goyang, Korea	2013-2015	retrospective	inoperable or recurrent HCC	71	63	PS 0: 100%	A: 68; B: 3	PSC	66 GyE/10 fr	91,3 Gy_10_	1.5cm (1.0–8.5)	31.3 mo (4.2–47)	3yr LPFS 89.9% (81.8–98%)	3yr 74.4% (63.1–85.7%)	Child–Pugh score 3pts 1-point increase.
**Kimura et al, 2017**	Radiation Oncology, Southern Tohoku Proton Beam Therapy Center	2008-2015	retrospective	HCC > 5 cm	24	73	PS 0:16; PS 1: 8	A: 100%	NA	72.6 GyE (60.8-85.8 GyE) 2.4 (2.2-6.6)Gy/fr	75 Gy_10_	5-18 CM	17.5 mo (3-64)	2yr 87%	2yr 52.4%	2G3 radiation dermatitis;
**Hong et al, 2016**	Multinstitutional	2009-2015	phase II prospective	HCC and ICC	44 HCC	70,5	PS 0:14, PS 1: 26, PS 2: 3	A:32, B:9, No cirrhosis: 3	PSC	58.0 Gy (40.5-67.5) in 15 FR	42.8 Gy_10_/81.5Gy_10_	5 cm (1.9-12.0)	All pts 19.5 months (0.6 - 55.9 months)	2yr 94.8%	1yr 76.5%, 2yr 63.2%	.1 G3 thrombocytopenia.
**Fukuda et al, 2016**	Proton Medical Research Center, Tsukuba, Japan	2002-2009	retrospective	previously untreated HCC	129	72	PS 0:70, PS 1:50, PS 2:9	A:101 B:28	PBS	54 pts 66GyE/10fx; 45 72.6GyE/22fx; 30 77GyE/35fx	91,3 Gy; 80,5 Gy; 78,3 Gy	3.9 cm(1–13.5)	55 mo (43-67)	5yr 94% (82-100%)	5-year OS rates were 69% (95% CI, 49–89%) for stage 0/A patients, 66% (95% CI, 48–84%) for stage B patients, and 25% (95% CI, 11–40%) for stage C patients	NONE
**Bush et al, 2016**	Loma Linda University Medical Center, Usa	NA	randomizedclinical trial: PT vs TACE	interim analysis report	33	61,4	N.A.	N.A.	PSC	70.2 CGE/15 fr	86 Gy_10_	3.2 cm(1.8-6.5)	28mo	2yr LC 88%	2yr OS 59%	2 pts requiring hospitalization for liver failure
**Kim et al, 2015**	National Cancer Center, Goyang, Korea	2007-2010	phase I	Dose finding	27	DL_1:70; DL_2:66; DL_3:63	PS 0:21; PS 1: 6	A: 24; B: 3	PSC	8 DL 1 60 GyE/20 fr; 7 DL 2 66 GyE/22 fr; 12 DL 3 72 GyE/24 fr	DL 1 65 Gy_10_ DL 2 71.5 Gy_10_ DL 3 80.2 Gy_10_	DL 1 3.2cm(2-7); DL 2 2.3cm(1.5-5); DL 3 2.5cm(1.3-6.2)	31 mo (5.2-63.4)	3yr LPFS 79.9% 5yr LPFS 63.9%	3yr 56.4% 5yr 42.3%	1 patient 1-point increase in CP score.
**Lee et al, 2014**	National Cancer Center, Goyang, Korea	2008-2011	retrospective	HCC with PVTT	27	55	PS 0:18; PS 1: 9	A: 18; B: 9	NA	median 55GyE (50–66 GyE)/20–22 fr	Max 71.5 Gy_10_	7cm (3–16)	13.2 mo (2.4–51.7)	1yr LPFS 70.7%; 2yr LPFS 61.9%	1yr 55.6%; 2yr 33.3%	4 pts 1-point increase in CP score.
**Sanford et al, 2019**	Massachusetts General Hospital, USA	2008/2017	retrospective	unresectable HCC	49	65	PS 0 23 pts; PS 1 24 pts, PS 2/3 2 pts	CP A 46 38 pts, CP B/C 8 pts	PSC	Various treatmetn schedules, the most adopted being 58GyE/15fr;	67 Gy_10_	NA	14mo (all pts)	2yr 93%	2 yr 59.1%	4 nonclassic RILD
**Hojo et al, 2019**	National Cancer Center Hospital East, Chiba, Japan	2008-2015	retrospective	anatomic subsegmental PT irradiation	110	74	PS 0 72 pts; PS 1-2 38 pts	CP A 95 pts, CP B 15 pts	PSC	76 Gy ()/20 fr	87.4 Gy10	74pts < 5 cm 36pts > 5 cm	36.5 mo (1-99.6 mo)	3yr 91.7%	3yr 74.2%	1 G3 radiation pneumonitis 1G3 liver dysfunction and 1 G3 bile duct stenosis. 1G5 radiation pneumonitis on day 188 after the start of PT
**Tamura et al, 2019**	Shizuoka Cancer Center Hospital, Japan	2003-2017	retrospective	single nodular HCC≤100 mm without vessel invasion	31	72	PS 0 20 pts; PS 1 10 pts, PS 2 1 pts	CP A 29 pts, CP B 2 pts	NA	8pts 66 GyE/10 Fr 22 pts 72.6–76 GyE/20–22 Fr 1pts 74–76 GyE/37–38 Fr	109.6 Gy10, 102.3 Gy10, 91.2 Gy10	3.5.Cm (1–9)	56.3 mo (22.2–82.3)	19.4% Local recurrence	3 yr 69.2%, 5yr 51.1%	1 G3 gastric ulcer
**Sekino et al, 2019**	Proton Medical Research Center, Tsukuba, Japan	2005-2014	retrospective	HCC pts with IVCTT	21	73	PS 0 12 pts; PS 1-3 9 pts	CP A 12 pts, CP B 9 pts	PBS	50–74 (median 72.6)	109.6 Gy10 median	8 cm (3.9–20)	21 mo (4–120)	100%	1yr OS 82%, 2yr OS 64%, 3 yr OS 36%	none
**Yoo et al, 2020**	Samsung Medical Center, Seoul, Korea	2016-2017	retrospective	compare the oncologic outcomes and toxicities between PS and PBS	172	62	PS 0 90 pts, PS 1-2 79 pts	CP A 154 pts, CP B 18 pts	133 PSC, 39 PBS	33 pts 50 Gy in 10 fractions 116 pts 60–66 Gy in 10 fractions 23 pts other schedules	75 Gy10, 109.6 Gy10, 132 Gy10	PS 3.1 (1–16); PBS 6.7 (1–19)	14 months (range, 1–31 months).	2-year 85.5%,	2-year OS 86.4%	RILD PS 2 pts PBS 1 patient 2 GI G3 (PS)
**Iwata et al, 2021**	Nagoya Proton Therapy Center, Japan	2013-2016	phase II	Operable or Ablation-Treatable HCC	45	68	PS 0 43 pts, PS 1 3 pts	CP A5 32, CP A6 9 pts, CP B7 4 pts	PSC	8 pts 72.6 Gy in 22 fr, 37 pts 66 Gy in 10 fr	96.6 Gy10, 109.6 Gy10	2.5 cm (1-10)	53 months (range, 9.5-75 months)	, 2yr LC 95%, 2yr PFS 62%	OS 5yr 70%	NO RILD, 1 G3 AST/ALT increase
**Iizumi et al, 2021**	Proton Medical Research Center, Tsukuba, Japan	2002-2014	retrospective	patients who received PBT for HCC in the caudate lobe	30	67	PS 0 12 pts, PS 1 17 pts, PS 2 1	CP A5 17 pts, CP A6 7 pts, CP B7 3 pts, CP B8 1 pts, CP C 1 pts, 1 pts N.A.	PSC	72.6 Gy in 22 fr (70%), 55 Gy in 10 fr (3.3%), 60 in 15 fr (3.3%), 74 in 37 fr (16.7%), 77 in 35 fr (6.7%)	96.6 Gy10, 85.2 Gy10, 84 Gy10, 88.8 Gy10, 93.9 Gy10	2.3 Cm (1-9)	37.5 months (3–152 months)	1yr LC 100%, 3yr LC 85.9%, 5yr LC 85.9%, 1yr PFS 65%, 3yr PFS 27.5%, 5yr PFS 22%	Os 1yr 86.6%, OS 3yr 62.8%, OS 5yr 46.1%	none
**Hsieh et al, 2019**	Chang Gung Memorial Hospital, Taiwan and MD Anderson Cancer Center, USA	2007-2017	retrospective	HCC who underwent definitive PT.	136	68	PS 0 68 pts, PS 1 64 pts, PS 2 4 pts	CP A5 92 pts, CP A6 25 pts, CP B7 13 pts, CP B8 6 pts	NA	72.6 Gy/22 fr ; 66 Gy/10 fr; 67,5 Gy/15 fr; 58 Gy/15 fr; 66 Gy/20 fr	80.5 Gy10, 91.3 Gy10, 81.6 Gy10, 67 Gy10, 73.2 Gy10	Median 6.8 cm in the eastern and 6.4 cm in the western pts, respectively.	10 mo (range, 5-27 months) and 23 mo (range, 3-76 mo) for the eastern and western patients, respectively	N.A.	N.A.	19 (14%) pts developed RILD
**Chiba et al, 2005**	Proton Medical Research Center, University of Tsukuba.	1985-1998	retrospective	pts unfit for surgery	162	62,5	PS 0 61; PS 1 79; PS 2 21;	CP A 82 CP B 62 CP C 10	PSC	72 Gy/16 fr 64 courses 78 Gy/20 fr 11 courses 84 Gy/20 fr 10 courses 50 Gy/10 fr 10 courses Miscellanous regimens 97 courses	87 Gy_10_; 90,4 Gy_10_; 94,5 Gy_10_; 62,5 Gy_10_	3 - 5 mm (108 pts)	31,7 months (3,1 to 133,2)	5 yr 89,6%	5 yr : 23,5%	Elevation of transaminase level 9,7%; Thrombocytopenia 3.2%.
**Komatsu et al, 2011**	Hyogo Ion Beam Medical Center, Tatsuno, Japan	2001-2009	Retrospective	All pts treated with PT for HCC	242	< 70 115 pts ≥ 70 127 pts	PS 0 172; PS 1 57; PS2 10 PS 3 3	CP A 184 CP B 55 CP C 3	PSC	76 Gy/20 Fr 70 pts 60 Gy/10 Fr 89 pts 66 Gy/10 Fr 53 pts miscellanous regimens 30 pts	104.8Gy_10_ 96 Gy_10_ 109.5 Gy_10_	< 5 cm 196 5-10 cm 67 > 10 cm 17	31 mo	90.2% at 5 yr	38% at 5yr	1G4 dermatitis 4G3 dermatitis 1 G3 Elevation of transaminase level 1G3 GI ulcer 1 G3 biloma
**Kawashima et al 2005**	National Cancer Center Hospital East, Chiba, Japan	1999-2003	Propsective	Phase II study	30	70	PS 0-1 29 PS 21	CP A 20 CP B 10	PSC	76 Gy/20 Fr	104.8Gy_10_	4.5 cm	31 mo	96% at 2 yr	66% at 2yr	5 G3 Elevation of transaminase level 5 G3 Leukopenia 7 G3 Thrombocytopenia 1 G3 bilirubine 8 pts developed PHI

PSC, passive scattering; PBS, pencil beam scanning; PS, performance status; CP, Child-Pugh; pts, patients. GI, gastrointestinal; PHI, proton-inducing hepatic insufficiency; mo, months; yr, years. Refer to the text for othe abbreviations.

In the USA, the pivotal prospective study from the University of Loma Linda by Bush et al. established the effectiveness of PT treatment for HCC in Western countries (33). The adopted 15-fraction treatment up to 63 Gy schedule gave positive results in terms of efficacy and safety and became the backbone of the multicenter, prospective phase II study by Hong et al. (34), published in 2016, whose positive findings led to the insertion of PT as an alternative treatment option in the NCCN guideline for unresectable HCC. The study evaluated 81 patients (44 HCC and 37 intrahepatic cholangiocarcinomas); the dose regimen was 67.5 Gy in 15 fractions for peripheral tumors and 58.05 Gy in 15 fractions for lesions within 2 cm of the porta hepatis. The 2-year LC, PFS, and OS rates in the HCC population were 94.8%, 39.9%, and 63.2%, respectively, with only one patient in the HCC cohort developing G3 toxicity (thrombocytopenia). More recently, several other reports from different institutions in the USA and Eastern countries confirmed the safety and effectiveness of PT in the treatment of HCC (**Table 1**). Parzen et al. in 2020 evaluated the multi-institutional prospective proton registry database and identified 30 HCC patients treated at nine institutions in the USA between 2013 and 2019 (35); the LC at 1 year was 91.2%, comparable with the historical series. A trend toward a statistically significant association between the BED and local control was observed.

Based on the recent reports from the Eastern countries, in addition to the already mentioned randomized trial of PT vs. ablation, the work of Kim et al. in 2019 retrospectively analyzed a large cohort (*n* = 243) of HCC patients treated at their institution with a risk-adapted treatment strategy according to the proximity of target to the gastrointestinal tract. Patients were treated with three different dose schedules in 10 fractions using the simultaneous boost technique to reduce the dose within 2 cm of the gastrointestinal tract; a significant association with the dose fractionation scheme, total PT dose, and OS was found (36).

## Technical challenges

Protons and, more in general, charged hadrons have the unique physical property of a finite range in tissues. The range is determined by the initial energy of the proton beam and by the stopping power of the material in the beam path.

This characteristic gives the possibility to obtain very high conformal dose distributions and the possibility to lower the mean and low dose bath around the target. The majority of clinical data depended on the passive scattering (PS) delivery method. New PT installations are mainly equipped with the pencil beam scanning (PBS) delivery technique. With PBS, given the possibility to modulate the intensity of the beam, higher dose conformity can be achieved with respect to PS at the cost of being more sensitive to uncertainties.

As a matter of fact, the quality of the nominal dose distribution can be perturbed by different sources of uncertainties like setup uncertainties, daily anatomical variations, uncertainty in machine delivery parameters, tissue inhomogeneity, inaccuracies in dose calculation algorithm, inaccuracies in CT calibration curve, and perturbations induced by internal organ motion (37). Every time a moving target is treated, the combination of its motion with an active delivery technique (such as proton pencil beam scanning, intensity-modulated radiation therapy, and volumetric arc therapy) can lead to an undesired deterioration of the dose distribution. This is the interplay effect. The management of this kind of uncertainty in particle therapy was widely discussed in an AAPM report recently published (38), and a comprehensive review on the clinical necessity of adequate imaging taking into account this effect has been published (39). The evaluation of interplay effects is the main technical challenge for the commissioning of liver treatments in free breathing and/or in breath-hold (40, 41). Different methods have been proposed to mitigate the effect of the motion on the dose distribution such as repainting (42), 4D optimization taking into account the organ motion during the planning (43), both 4D optimization and repainting (44), motion reduction with the use of compressor (45), or forced deep expiration breath-hold (46, 47).

The setup uncertainty has another crucial role in the treatment of liver tumors (48). In particular, a comparison between vertebral body matching, diaphragm matching, and marker matching has been analyzed concluding that the last one has the best results in terms of positioning accuracy. Consideration has to be made from a radiobiological point of view if radio-opaque markers larger than 1.5 mm are used in the context of particle therapy since they can reduce the tumor control probability (TCP) and increase the dose to the surrounding critical organs (49). The diaphragm matching can be a reliable surrogate for liver tumor alignment (50). A detailed technical report of the first 17 liver patients treated with forced deep expiration breath has been reported by Fracchiolla et al. (51). The use of the Active Breathing Coordinator (ABC-ELEKTA^®^) reduced the residual motion of the internal organs during the delivery and increased the reproducibility of the patient anatomy. The authors also proposed a method to optimize the use of the range shifter in order to obtain a sharper lateral dose penumbra and, for facilities with more than a single treatment room, to optimize the beam time allocation.

## Future directions

There is growing scientific evidence regarding the effectiveness and safety of the use of PT for HCC. In recent years, the strength of evidence increased, with several data coming from prospective studies and also from two randomized studies. **Table 2** illustrates the clinical trials currently evaluating the use of protons for HCC. The superiority of PT in comparison with X-rays in terms of OS for HCC patients, which has been highlighted so far only in retrospective studies, will be evaluated in the already mentioned multicenter trial NCT03186898. The Mayo Clinic phase II trial has the goal to evaluate the safety of the use of 5-fraction stereotactic PT for the treatment of HCC. Another interesting trial is the NCT05203120 that is currently ongoing at the Danish Particle Center in Denmark, the first European prospective study for PT and HCC, whose results, if positive, could help in bridging the gap between Europe and the USA and Eastern countries in acknowledging the effectiveness of radiotherapy in the treatment of HCC. Other strategies to evaluate in the next future studies should include the combination of PT with other locoregional therapies. The positive results of the already mentioned IMbrave150 phase III trial demonstrated the effectiveness of combination therapy for advanced stage HCC. In the context of locoregional disease, the combination of local and locoregional therapy such as TACE and radiotherapy could have an impact on the oncological outcome, probably at a cost of higher toxicity, as reported by the meta-analysis by Meng et al. (52). The favorable toxicity profile of protons due to their intrinsic physical properties makes PT the option of choice in case of combined treatments, especially for complex settings such as large tumors and poor liver function. Furthermore, the combination of PT with immune checkpoint inhibitors, which has been recently retrospectively reported (53) (**Table 1**), should be evaluated in prospective trials for safety and effectiveness.

**Table 2:** Clinical Trials currently open evaluating PT for HCC.

Study Title	Principal Institution	Type of trial	ClinicalTrials.gov Identifier	Estimated Enrollment	Actual Study Start Date	Estimated Study Completion Date
Feasibility of High Dose Proton Therapy On Unresectable Primary Carcinoma Of Liver: Prospective Phase II trial	Samsung Medical Center, Seoul, Korea	Monoinstitutional, Phase II	NCT02632864	66 participants	2015	2022
Proton Beam Therapy in Hepatocellular Carcinoma With Portal Vein Tumor Thrombosis (PTHP)	Samsung Medical Center, Seoul, Korea	Monoinstitutional, Phase II	NCT02571946	53 participants	2015	2022
Radiation Therapy With Protons or Photons in Treating Patients With Liver Cancer	NRG Oncology Massachusetts General Hospital Cancer Center, USA	Multicentric, Phase III	NCT03186898	186 participants	2017	2029
Stereotactic Body Proton Radiotherapy for the Treatment of Liver Cancer	Mayo Clinic, USA	Multicentric, Phase II	NCT04805788	60 participants	2021	2025
A National Phase II Study of Proton Therapy in Hepatocellular Carcinoma	Aarhus University Hospital, Denmark	Multicentric, Phase II	NCT05203120	50 participants	2022	2030

## Author contributions

FD, AB, and GS: manuscript conception and design of the study. FD, DS, FF, LG, BS, MC, GS, IG and AB: writing of the manuscript. All authors contributed to the article and approved the submitted version.

## Funding

The authors declare that this study received funding from Azienda Provinciale per i Servizi Sanitari. The funder was not involved in the study design, collection, analysis, interpretation of data, the writing of this article or the decision to submit it for publication.

## Conflict of interest

The authors declare that the research was conducted in the absence of any commercial or financial relationships that could be construed as a potential conflict of interest.

## Publisher’s note

All claims expressed in this article are solely those of the authors and do not necessarily represent those of their affiliated organizations, or those of the publisher, the editors and the reviewers. Any product that may be evaluated in this article, or claim that may be made by its manufacturer, is not guaranteed or endorsed by the publisher.

## References

[1] Cancer today (2022). Available at: http://gco.iarc.fr/today/home.

[2] Bray F Ferlay J Soerjomataram I Siegel RL Torre LA Jemal A. Global cancer statistics 2018: GLOBOCAN estimates of incidence and mortality worldwide for 36 cancers in 185 countries. CA Cancer J Clin (2018) 68(6):394–424. doi: 10.3322/caac.21492 30207593

[3] Trevisani F Cantarini MC Wands JR Bernardi M. Recent advances in the natural history of hepatocellular carcinoma. Carcinogenesis (2008) 29(7):1299–305. doi: 10.1093/carcin/bgn113 18515282

[4] Llovet JM Bustamante J Castells A Vilana R Ayuso M del C Sala M. Natural history of untreated nonsurgical hepatocellular carcinoma: rationale for the design and evaluation of therapeutic trials. Hepatology (1999) 29(1):62–7. doi: 10.1002/hep.510290145 9862851

[5] Jiang YQ Wang ZX Deng YN Yang Y Wang GY Chen GH. Efficacy of hepatic resection vs. radiofrequency ablation for patients with very-Early-Stage or early-stage hepatocellular carcinoma: A population-based study with stratification by age and tumor size. Front Oncol (2019) 9:113. doi: 10.3389/fonc.2019.00113 30863723 PMC6400103

[6] Llovet JM Bruix J. Systematic review of randomized trials for unresectable hepatocellular carcinoma: Chemoembolization improves survival. Hepatology (2003) 37(2):429–42. doi: 10.1053/jhep.2003.50047 12540794

[7] Salem R Gordon AC Mouli S Hickey R Kallini J Gabr A. Y90 radioembolization significantly prolongs time to progression compared with chemoembolization in patients with hepatocellular carcinoma. Gastroenterology (2016) 151(6):1155–63.e2. doi: 10.1053/j.gastro.2016.08.029 27575820 PMC5124387

[8] Finn RS Qin S Ikeda M Galle PR Ducreux M Kim TY. Atezolizumab plus bevacizumab in unresectable hepatocellular carcinoma. N Engl J Med (2020) 382(20):1894–905. doi: 10.1056/NEJMoa1915745 32402160

[9] Llovet JM Ricci S Mazzaferro V Hilgard P Gane E Blanc JF. Sorafenib in advanced hepatocellular carcinoma. N Engl J Med (2008) 359(4):378–90. doi: 10.1056/NEJMoa0708857 18650514

[10] Lievens Y Ricardi U Poortmans P Verellen D Gasparotto C Verfaillie C. Radiation oncology. optimal health for all, together. ESTRO vision, 2030. Radiother Oncol (2019) 136:86–97. doi: 10.1016/j.radonc.2019.03.031 31015134

[11] Brock KK. Imaging and image-guided radiation therapy in liver cancer. Semin Radiat Oncol (2011) 21(4):247–55. doi: 10.1016/j.semradonc.2011.05.001 PMC3428059 21939853

[12] Bujold A Massey CA Kim JJ Brierley J Cho C Wong RKS. Sequential phase I and II trials of stereotactic body radiotherapy for locally advanced hepatocellular carcinoma. J Clin Oncol (2013) 31(13):1631–9. doi: 10.1200/JCO.2012.44.1659 23547075

[13] Jang WI Kim MS Bae SH Cho CK Yoo HJ Seo YS. High-dose stereotactic body radiotherapy correlates increased local control and overall survival in patients with inoperable hepatocellular carcinoma. Radiat Oncol (2013) 8:250. doi: 10.1186/1748-717X-8-250 24160944 PMC4231524

[14] Park S Yoon WS Rim CH. Indications of external radiotherapy for hepatocellular carcinoma from updated clinical guidelines: Diverse global viewpoints. World J Gastroenterol (2020) 26(4):393–403. doi: 10.3748/wjg.v26.i4.393 32063688 PMC7002906

[15] PTCOG. Facilities in operation (2022). Available at: https://www.ptcog.ch/index.php/facilities-in-operation.

[16] Dawson LA Normolle D Balter JM McGinn CJ Lawrence TS Ten Haken RK. Analysis of radiation-induced liver disease using the Lyman NTCP model. Int J Radiat Oncol Biol Phys (2002) 53(4):810–21. doi: 10.1016/S0360-3016(02)02846-8 12095546

[17] Pan CC Kavanagh BD Dawson LA Li XA Das SK Miften M. Radiation-associated liver injury. Int J Radiat Oncol Biol Phys (2010) 76(3 Suppl):S94–100. doi: 10.1016/j.ijrobp.2009.06.092 20171524 PMC4388033

[18] Wang X Krishnan S Zhang X Dong L Briere T Crane CH. Proton radiotherapy for liver tumors: dosimetric advantages over photon plans. Med Dosim (2008) 33(4):259–67. doi: 10.1016/j.meddos.2007.04.008 18973852

[19] Qi WX Fu S Zhang Q Guo XM. Charged particle therapy versus photon therapy for patients with hepatocellular carcinoma: a systematic review and meta-analysis. Radiother Oncol (2015) 114(3):289–95. doi: 10.1016/j.radonc.2014.11.033 25497556

[20] Sanford NN Pursley J Noe B Yeap BY Goyal L Clark JW. Protons versus photons for unresectable hepatocellular carcinoma: Liver decompensation and overall survival. Int J Radiat Oncol Biol Phys (2019) 105(1):64–72. doi: 10.1016/j.ijrobp.2019.01.076 30684667

[21] Cheng JY Liu CM Wang YM Hsu HC Huang EY Huang TT. Proton versus photon radiotherapy for primary hepatocellular carcinoma: a propensity-matched analysis. Radiat Oncol (2020) 15(1):159. doi: 10.1186/s13014-020-01605-4 32605627 PMC7325065

[22] Rawlins M. De testimonio: on the evidence for decisions about the use of therapeutic interventions. Clin Med (Lond) (2008) 8(6):579–88. doi: 10.1016/S0140-6736(08)61930-3 PMC4954394 19149278

[23] Forner A Llovet JM Bruix J. Hepatocellular carcinoma. Lancet (2012) 379(9822):1245–55. doi: 10.1016/S0140-6736(11)61347-0 PMC13537732 22353262

[24] Bush DA Smith JC Slater JD Volk ML Reeves ME Cheng J. Randomized clinical trial comparing proton beam radiation therapy with transarterial chemoembolization for hepatocellular carcinoma: Results of an interim analysis. Int J Radiat Oncol Biol Phys (2016) 95(1):477–82. doi: 10.1016/j.ijrobp.2016.02.027 27084661

[25] Tamura S Okamura Y Sugiura T Ito T Yamamoto Y Ashida R. A comparison of the outcomes between surgical resection and proton beam therapy for single primary hepatocellular carcinoma. Surg Today (2019) 50(4):369–78. doi: 10.1007/s00595-019-01888-5 31602531

[26] Kim TH Koh YH Kim BH Kim MJ Lee JH Park B. Proton beam radiotherapy vs. radiofrequency ablation for recurrent hepatocellular carcinoma: A randomized phase III trial. J Hepatol (2021) 74(3):603–12. doi: 10.1016/j.jhep.2020.09.026 33031846

[27] Dionisi F Brolese A Siniscalchi B Giacomelli I Fracchiolla F Righetto R. Clinical results of active scanning proton therapy for primary liver tumors. Tumori (2021) 107(1):71–9. doi: 10.1177/0300891620937809 32648818

[28] Nakayama H Sugahara S Tokita M Fukuda K Mizumoto M Abei M. Proton beam therapy for hepatocellular carcinoma: the university of tsukuba experience. Cancer (2009) 115(23):5499–506. doi: 10.1002/cncr.24619 19645024

[29] Sugahara S Oshiro Y Nakayama H Fukuda K Mizumoto M Abei M. Proton beam therapy for large hepatocellular carcinoma. Int J Radiat Oncol Biol Phys (2010) 76(2):460–6. doi: 10.1016/j.ijrobp.2009.02.030 19427743

[30] Hata M Tokuuye K Sugahara S Kagei K Igaki H Hashimoto T. Proton beam therapy for hepatocellular carcinoma with portal vein tumor thrombus. Cancer (2005) 104(4):794–801. doi: 10.1002/cncr.21237 15981284

[31] Hata M Tokuuye K Sugahara S Tohno E Nakayama H Fukumitsu N. Proton beam therapy for aged patients with hepatocellular carcinoma. Int J Radiat Oncol Biol Phys (2007) 69(3):805–12. doi: 10.1016/j.ijrobp.2007.04.016 17524568

[32] Hata M Tokuuye K Sugahara S Fukumitsu N Hashimoto T Ohnishi K. Proton beam therapy for hepatocellular carcinoma patients with severe cirrhosis. Strahlenther Onkol (2006) 182(12):713–20. doi: 10.1007/s00066-006-1564-2 PMC3233367 17149578

[33] Bush DA Kayali Z Grove R Slater JD. The safety and efficacy of high-dose proton beam radiotherapy for hepatocellular carcinoma: a phase 2 prospective trial. Cancer (2011) 117(13):3053–9. doi: 10.1002/cncr.25809 21264826

[34] Hong TS Wo JY Yeap BY Ben-Josef E McDonnell EI Blaszkowsky LS. Multi-institutional phase II study of high-dose hypofractionated proton beam therapy in patients with localized, unresectable hepatocellular carcinoma and intrahepatic cholangiocarcinoma. J Clin Oncol (2016) 34(5):460–8. doi: 10.1200/JCO.2015.64.2710 PMC4872014 26668346

[35] Parzen JS Hartsell W Chang J Apisarnthanarax S Molitoris J Durci M. Hypofractionated proton beam radiotherapy in patients with unresectable liver tumors: multi-institutional prospective results from the proton collaborative group. Radiat Oncol (2020) 15(1):255. doi: 10.1186/s13014-020-01703-3 33148296 PMC7643436

[36] Kim TH Park JW Kim BH Kim H Moon SH Kim SS. Does risk-adapted proton beam therapy have a role as a complementary or alternative therapeutic option for hepatocellular carcinoma? Cancers (Basel) (2019) 11(2):E230. doi: 10.3390/cancers11020230 30781391 PMC6406298

[37] Ribeiro CO Meijers A Korevaar EW Muijs CT Both S Langendijk JA. Comprehensive 4D robustness evaluation for pencil beam scanned proton plans. Radiother Oncol (2019) 136:185–9. doi: 10.1016/j.radonc.2019.03.037 31015123

[38] Li H Dong L Bert C Chang J Flampouri S Jee KW. AAPM task group report 290: Respiratory motion management for particle therapy. Med Phys (2022) 49(4):e50–81. doi: 10.1002/mp.15470 PMC9306777 35066871

[39] Knopf AC Czerska K Fracchiolla F Graeff C Molinelli S Rinaldi I. Clinical necessity of multi-image based (4DMIB) optimization for targets affected by respiratory motion and treated with scanned particle therapy - a comprehensive review. Radiother Oncol (2022) 169:77–85. doi: 10.1016/j.radonc.2022.02.018 35189152

[40] Zhang Y Boye D Tanner C Lomax AJ Knopf A. Respiratory liver motion estimation and its effect on scanned proton beam therapy. Phys Med Biol (2012) 57(7):1779–95. doi: 10.1088/0031-9155/57/7/1779 22407290

[41] Zhang Y Huth I Weber DC Lomax AJ. A statistical comparison of motion mitigation performances and robustness of various pencil beam scanned proton systems for liver tumour treatments. Radiother Oncol (2018) 128(1):182–8. doi: 10.1016/j.radonc.2018.01.019 29459153

[42] Zhang Y Huth I Wegner M Weber DC Lomax AJ. An evaluation of rescanning technique for liver tumour treatments using a commercial PBS proton therapy system. Radiother Oncol (2016) 121(2):281–7. doi: 10.1016/j.radonc.2016.09.011 27726957

[43] Pfeiler T Bäumer C Engwall E Geismar D Spaan B Timmermann B. Experimental validation of a 4D dose calculation routine for pencil beam scanning proton therapy. Z Med Phys (2018) 28(2):121–33. doi: 10.1016/j.zemedi.2017.07.005 28843397

[44] Siregar H Bäumer C Blanck O Chan M Engwall E Plaude S. Mitigation of motion effects in pencil-beam scanning - impact of repainting on 4D robustly optimized proton treatment plans for hepatocellular carcinoma. Z Med Phys (2022) 32(1):63–73. doi: 10.1016/j.zemedi.2020.08.001 33131995 PMC9948857

[45] Lin L Souris K Kang M Glick A Lin H Huang S. Evaluation of motion mitigation using abdominal compression in the clinical implementation of pencil beam scanning proton therapy of liver tumors. Med Phys (2017) 44(2):703–12. doi: 10.1002/mp.12040 28133755

[46] Fracchiolla F Dionisi F Giacomelli I Hild S Esposito PG Lorentini S. Implementation of proton therapy treatments with pencil beam scanning of targets with limited intrafraction motion. Phys Med (2019) 57:215–20. doi: 10.1016/j.ejmp.2019.01.007 30661743

[47] Apisarnthanarax S Saini J O’Ryan-Blair A Castro J Bowen SR. Intensity modulated proton therapy with advanced planning techniques in a challenging hepatocellular carcinoma patient. Cureus (2017) 9(9):e1674. doi: 10.7759/cureus.1674 29152431 PMC5679776

[48] Takemasa K Kato T Narita Y Kato M Yamazaki Y Ouchi H. The impact of different setup methods on the dose distribution in proton therapy for hepatocellular carcinoma. J Appl Clin Med Phys (2021) 22(3):63–71. doi: 10.1002/acm2.13178 PMC7984466 33595910

[49] Matsuura T Maeda K Sutherland K Takayanagi T Shimizu S Takao S. Biological effect of dose distortion by fiducial markers in spot-scanning proton therapy with a limited number of fields: a simulation study. Med Phys (2012) 39(9):5584–91. doi: 10.1118/1.4745558 22957624

[50] Yang J Cai J Wang H Chang Z Czito BG Bashir MR. Is diaphragm motion a good surrogate for liver tumor motion? Int J Radiat Oncol Biol Phys (2014) 90(4):952–8. doi: 10.1016/j.ijrobp.2014.07.028 PMC4334361 25223297

[51] Fracchiolla F Dionisi F Righetto R Widesott L Giacomelli I Cartechini G. Clinical implementation of pencil beam scanning proton therapy for liver cancer with forced deep expiration breath hold. Radiother Oncol (2021) 154:137–44. doi: 10.1016/j.radonc.2020.09.035 32976870

[52] Meng MB Cui YL Lu Y She B Chen Y Guan YS. Transcatheter arterial chemoembolization in combination with radiotherapy for unresectable hepatocellular carcinoma: a systematic review and meta-analysis. Radiother Oncol (2009) 92(2):184–94. doi: 10.1016/j.radonc.2008.11.002 19042048

[53] Su CW Hou MM Huang PW Chou YC Huang BS Tseng JH. Proton beam radiotherapy combined with anti-PD1/PDL1 immune checkpoint inhibitors for advanced hepatocellular carcinoma. Am J Cancer Res (2022) 12(4):1606–20. PMC9077059 35530291

